# Natal factors affecting developmental defects of enamel in preterm infants: a prospective cohort study

**DOI:** 10.1038/s41598-024-52525-2

**Published:** 2024-01-24

**Authors:** Shang-yon Park, Su Jin Jeong, Jung Ho Han, Jeong Eun Shin, Jae-Ho Lee, Chung-Min Kang

**Affiliations:** 1 Department of Pediatric Dentistry, Yonsei University College of Dentistry, Seoul, Republic of Korea; 2 Statistics Support Part, Medical Science Research Institute, Kyung Hee University Medical Center, Seoul, Republic of Korea; 3 Department of Pediatrics, Yonsei University College of Medicine, Seoul, Republic of Korea; 4 Oral Science Research Center, Yonsei University College of Dentistry, Seoul, Republic of Korea

**Keywords:** Risk factors, Paediatric research

## Abstract

This study investigated natal factors influencing developmental defects of enamel (DDE) in premature infants using a newly refined preterm developmental defects of enamel (PDDE) index. Dental examinations were conducted on a cohort of 118 preterm infants (average age 3.5 ± 1.4 years) to record PDDE scores, while reviewing their medical records to examine natal factors. According to the logistic regression analysis, factors related to DDE prevalence were advanced maternal age, gestational age < 28 weeks, birth weight < 1000 g, 1 min APGAR score < 7, and hospitalization period > 2 months, which were significantly higher by 2.91, 5.53, 7.63, 10.02, and 4.0 times, respectively. According to regression analysis with generalized linear models, the PDDE scores were approximately 7.65, 4.96, and 15.0 points higher in premature children diagnosed with bronchopulmonary dysplasia, intraventricular hemorrhage, and necrotizing enterocolitis, respectively. When endotracheal intubation was performed, the PDDE score was 5.06 points higher. The prevalence of PDDE was primarily observed bilaterally in the maxillary anterior teeth. Extremely preterm infants showed significantly delayed tooth eruption, suggesting that the influence of gestational age on dental development rates. Identifying the factors related to DDE in premature children can inform early dental interventions to support the oral health of high-risk children.

## Introduction

Preterm birth is defined as babies born before 37 weeks of pregnancy and are classified into three subcategories based on gestational age (GA) according to the World Health Organization: extremely preterm (GA < 28 weeks), very preterm (28 ≤ GA < 32 weeks), and moderate to late preterm (32 ≤ GA < 37 weeks). Premature infants are vulnerable to various complications owing to their underdeveloped physiological systems^1^. Medical progress has improved preterm infant survival; however, it has also increased the incidence of severe complications, signifying the need for medical care and dental treatment for preterm infants^2,3^.

As tooth enamel lacks a natural repair mechanism, disruption of amelogenesis can lead to permanent defect in the enamel^4^. Enamel formation begins at 4 months of gestation and continues until 11 months after birth, making it susceptible to pre-, neo-, and postnatal conditions^4–9^. Consequently, preterm infants may experience abnormal tooth color and morphological irregularities. Developmental defects of enamel (DDE) are defined as abnormalities in the quantity and quality of dental enamel^10,11^. Enamel hypoplasia refers to a quantitative defect characterized by enamel deficiency, whereas enamel hypomineralization represents a qualitative defect that involves diffuse, demarcated, or white, yellow, or brown discoloration with increasing severity as the color darkens^11,12^. Enamel hypoplasia and hypomineralization occur when abnormalities occur during the secretion and maturation phase, respectively^13^.

The higher prevalence of DDE in preterm and low-birthweight (BW) infants has been well-established, ranging from 1.33 to 9.87 times greater than that in full-term infants^14–16^. The association between systemic complications and DDE have also been explored^5,9,17–24^. Infants with intrauterine growth restriction (IUGR) exhibited a 5.19-fold higher prevalence of DDE^5^. Bronchopulmonary dysplasia (BPD) and necrotizing enterocolitis (NEC), which disturb mineral equilibrium or impede mineral absorption, have also been theorized to be linked with DDE^19–21^. However, previous research has several limitations, such as small sample sizes and a lack of medical variables^5,20^, focusing only on the correlation with a lack of assessment of DDE severity^5,9,17–24^.

The objective of this study was to investigate various factors that may influence prevalence and severity of DDE in preterm infants. Additionally, it proposed a new index (preterm developmental defects of enamel, PDDE) that enables severity assessment of qualitative and quantitative defects separately. The severity of qualitative defects is assessed based on tooth color and opacity, while that of quantitative defects is assessed based on the extent of hard tissue defects.

## Results

This study included 118 preterm infants: 60 (50.8%) males and 58 (49.2%) females. Preterm infants were divided into three groups: extremely preterm (n = 43), very preterm (n = 30), and moderate to late preterm (n = 45). The case records were completed at an average age of 3.5 ± 1.4 years, at which point all 20 primary teeth had erupted. The mean GA of the preterm infants was 30.3 ± 4.1 weeks, and the mean BW was 1408 ± 677.7 g. Significant differences in BW, 1- and 5-min APGAR score were observed among each group (*p* < 0.0001), all of which decreased as GA decreased. A shorter GA was associated with an increased incidence of medical complications (BPD, rickets, intraventricular hemorrhage (IVH), NEC, and IUGR). Demographic data of the study groups (n = 118) are presented in Table 1.

**Table 1:** Demographics of the study population based on gestational age (GA).

Characteristics	All (n = 118)	GA < 28 (n = 43)	28 ≤ GA < 32 (n = 30)	32 ≤ GA < 37 (n = 45)	P-value
Prenatal factors					
Maternal age (year)^a^	34.6 (± 4.4)	34.8 (± 4.3)	34.2 (± 5.3)	34.7 (± 3.9)	0.8761
Paternal age (year)^a^	36.6 (± 5.1)	36.4 (± 5.0)	35.7 (± 5.0)	37.0 (± 5.3)	0.7285
Maternal abortion history	27 (31.0)	7 (25.0)	3 (12.5)	17 (48.6)	0.0093*
Neonatal factors					
Sex (male:female)	60:58 (50.8:49.2)	20:23 (46.5:53.5)	13:17 (43.3:56.7)	27:18 (60:40)	0.2852
Birth weight (g)^a^	1408.1 (± 677.9)	831.5 (± 148.6)	1180.2 (± 363.8)	2110.9 (± 498.3)	< 0.0001*
APGAR score^a^					
1 min	4.2 (± 1.8)	3.1 (± 1.1)	4.0 (± 1.8)	5.3 (± 1.5)	< 0.0001*
5 min	6.1 (± 1.7)	5.1 (± 1.4)	5.8 (± 1.4)	7.2 (± 1.4)	< 0.0001*
Delivery mode (C-sec:NSVD)	97:20 (82.9:17.1)	37:5 (88.1:11.9)	26:4 (86.7:13.3)	34:11 (75.6:24.4)	0.2450
Multiple pregnancy (Singlet:Multiplet)	73:44 (62.4:37.6)	26:16 (61.9:38.1)	21:9 (70.0:30.0)	26:19 (57.8:42.2)	0.5620
Postnatal factors					
Medical complications					
Bronchopulmonary dysplasia	66 (57.9)	40 (93.0)	23 (88.5)	3 (6.7)	< 0.0001*
Ricket	27 (23.7)	18 (41.9)	6 (23.1)	3 (6.7)	0.0005*
Intraventricular hemorrhage	40 (35.1)	23 (53.5)	9 (34.6)	8 (17.8)	0.0021*
Necrotizing enterocolitis	13 (11.4)	10 (23.3)	2 (7.7)	1 (2.2)	0.0064*
Hyperbilirubinemia	73 (64.0)	24 (55.8)	17 (65.4)	32 (71.1)	0.3229
Intrauterine growth restriction	21 (18.9)	3 (7.3)	3 (11.1)	15 (34.9)	0.0027*
Sepsis	31 (27.2)	14 (32.6)	6 (23.1)	11 (24.4)	0.6007
Hypocalcemia	18 (15.8)	6 (14.0)	5 (19.2)	7 (15.6)	0.8426
Endotracheal intubation (days)^a^	20.3 (± 37.9)	32.9 (± 49.3)	24.7 (± 36.4)	6.2 (± 17.5)	< 0.0001*
Parenteral nutrition (days)^a^	25.6 (± 41.9)	51.3 (± 55.6)	26.1 (± 34.3)	5.1 (± 12.5)	< 0.0001*
NICU hospitalization (days)^a^	92.0 (± 71.2)	150.4 (± 62.0)	93.4 (± 52.0)	39.3 (± 42.4)	< 0.0001*
Tooth eruption age					
Chronological age (months)^a^	7.93 (± 3.16)	8.07 (± 3.46)	8.58 (± 3.22)	7.28 (± 2.73)	0.5853
Corrected age (months)^a^	10.62 (± 4.42)	12.17 (± 5.48)	11.16 (± 3.35)	8.40 (± 2.61)	0.0016*

Values are presented as average (± SD) or number (%). For categorical data, the p-value was tested using the chi-squared test. For the numerical data, the *p*-value was tested using the Kruskal–Wallis test. Bonferroni post hoc tests were performed to conduct multiple pairwise comparisons after the initial analyses. *p < 0.05 indicates statistical significance. ^a^ indicates the data tested using the Kruskal–Wallis test.

*C-sec* cesarean section; *NSVD* normal spontaneous vaginal delivery; *NICU* neonatal intensive care unit.

### Regression analysis for prevalence of DDE in preterm infants

Advanced maternal age was associated with an increased prevalence of DDE (OR 2.91, *p* = 0.0494). Among the neonatal risk factors, extremely preterm infants (OR 5.53, *p* = 0.0214), BW < 1000 g (OR 7.63, *p* = 0.0154), 1-min APGAR score < 7 (OR 10.02, *p* = 0.0005), and 5-min APGAR score < 8 (OR 7.00, *p* = 0.0005) increased the prevalence of DDE. Among the postnatal factors, only the duration of neonatal intensive care unit (NICU) admission > 2 months significantly increased the prevalence of DDE (OR 4.00, *p* = 0.0097) (Table 2).

**Table 2:** Regression analysis of potential risk factors for increasing the prevalence of developmental defects on enamel prevalence.

Independent variable		Descriptive analysis				Logistic regression analysis		
		N	Normal	Defects	P-value	OR	95% CI	P-value
Prenatal risk factors								
Advanced maternal age	Yes	43	11 (25.6)	32 (74.4)	0.0584	2.91	1.00–8.46	0.0494*
	No	59	6 (10.2)	53 (89.8)		Reference		
Maternal abortion history	Yes	27	4 (14.8)	23 (85.2)	1.0000	0.85	0.23–2.94	0.7941
	No	60	8 (13.3)	52 (86.7)		Reference		
Neonatal risk factors								
Sex	Male	60	9 (15.0)	51 (85.0)	1.0000	1.04	0.39–2.80	0.9374
	Female	58	9 (16.0)	49 (84.0)		Reference		
Gestational age (GA, weeks)								
GA < 28		43	2 (4.7)	41 (95.3)	0.0275*	5.53	1.29–23.76	0.0214*
28 ≤ GA < 32		30	5 (16.7)	25 (83.3)		1.55	0.49–4.90	0.4599
32 ≤ GA < 37		45	11 (24.4)	34 (75.6)		Reference		
Birth weight (BW, g)								
BW < 1000		47	3 (6.4)	44 (93.6)	0.0516	7.63	1.48–39.43	0.0154*
1000 ≤ BW < 1500		25	4 (16.0)	21 (84.0)		2.87	0.58–14.21	0.1975
1500 ≤ BW < 2500		35	7 (20.0)	28 (80.0)		2.28	0.53–9.90	0.2714
2500 ≤ BW		11	4 (36.36)	7 (63.6)			Reference	
Delivery mode	C-sec	97	15 (15.5)	82 (84.5)	1.0000	1.06	0.29–3.89	0.9249
	NVSD	20	3 (15.0)	17 (85.0)		Reference		
APGAR score 1min^a^	< 7	95	11 (11.6)	84 (88.4)	0.0006	10.02	2.73–36.84	0.0005*
	≥ 7	12	7 (58.3)	5 (41.7)		Reference		
APGAR score 5min^a^	< 8	84	8 (9.52)	76 (90.5)	0.0005	7.00	2.36–20.81	0.0005*
	≥ 8	23	10 (43.5)	13 (56.5)		Reference		
Multiple pregnancy	Multiplet	73	8 (11.0)	65 (89.0)	0.1135	0.43	0.16–1.16	0.0957
	Singlet	44	10 (22.7)	34 (77.3)		Reference		
Postnatal risk factors								
Medical complications								
Bronchopulmonary dysplasia	Yes	66	7 (10.6)	59 (89.4)	0.1169	2.43	0.88–6.72	0.0861
	No	48	11 (22.9)	37 (77.1)		Reference		
Ricket	Yes	27	2 (7.41)	25 (92.6)	0.2336	2.35	0.56–9.82	0.2400
	No	87	16 (18.4)	71 (81.6)		Reference		
Intraventricular hemorrhage	Yes	40	5 (12.5)	35 (87.5)	0.2336	1.42	0.48–4.20	0.5291
	No	74	13 (17.6)	61 (82.4)		Reference		
Necrotizing enterocolitis	Yes	13	1 (7.7)	12 (92.3)	0.6886	1.73	0.28–10.78	0.5595
	No	101	17 (16.8)	84 (83.2)		Reference		
Hyperbilirubinemia	Yes	73	15 (20.5)	58 (79.5)	0.1060	0.34	0.10–1.18	0.0916
	No	41	3 (7.3)	38 (92.7)		Reference		
Intrauterine growth restriction	Yes	21	4 (19.0)	17 (81.0)	0.7441	0.74	0.22–2.45	0.6186
	No	90	14 (15.6)	76 (84.4)		Reference		
Hypocalcemia	Yes	18	2 (11.1)	16 (88.9)	0.7335	1.35	0.17–3.21	0.6860
	No	96	16 (16.7)	80 (83.3)		Reference		
Sepsis	Yes	31	7 (22.6)	24 (77.4)	0.2534	0.51	0.18–1.47	0.2154
	No	83	11 (13.3)	72 (86.7)		Reference		
Parenteral feeding (month)^a^	≥ 1	28	2 (7.14)	26 (92.9)	0.1398	2.91	0.69–12.23	0.1451
	< 1	71	15 (21.1)	56 (78.9)		Reference		
Period of intubation (month)^a^	≥ 1	23	1 (4.3)	22 (95.7)	0.1128	3.83	0.65–22.5	0.1366
	< 1	85	17 (20.0)	68 (80.0)		Reference		
Period of NICU admission (month)^a^	≥ 2	69	6 (8.70)	63 (91.3)	0.0081*	4.00	1.40–11.45	0.0097*
	< 2	42	12 (28.6)	30 (71.4)		Reference		

Values are presented as numbers (%). For descriptive analysis, *p*-value was tested using Fisher's exact test. For logistic regression, *p*-value was tested using Firth's method.

*C-sec* cesarean section; *NSVD* normal spontaneous vaginal delivery; *NICU* neonatal intensive care unit.

*p < 0.05 indicates statistical significance.

^a^Indicates that the cutoff value was determined by the receiver operating characteristic (ROC) curve.

### Medical complications attributing to increased PDDE score in preterm infants

**Figure 1:**
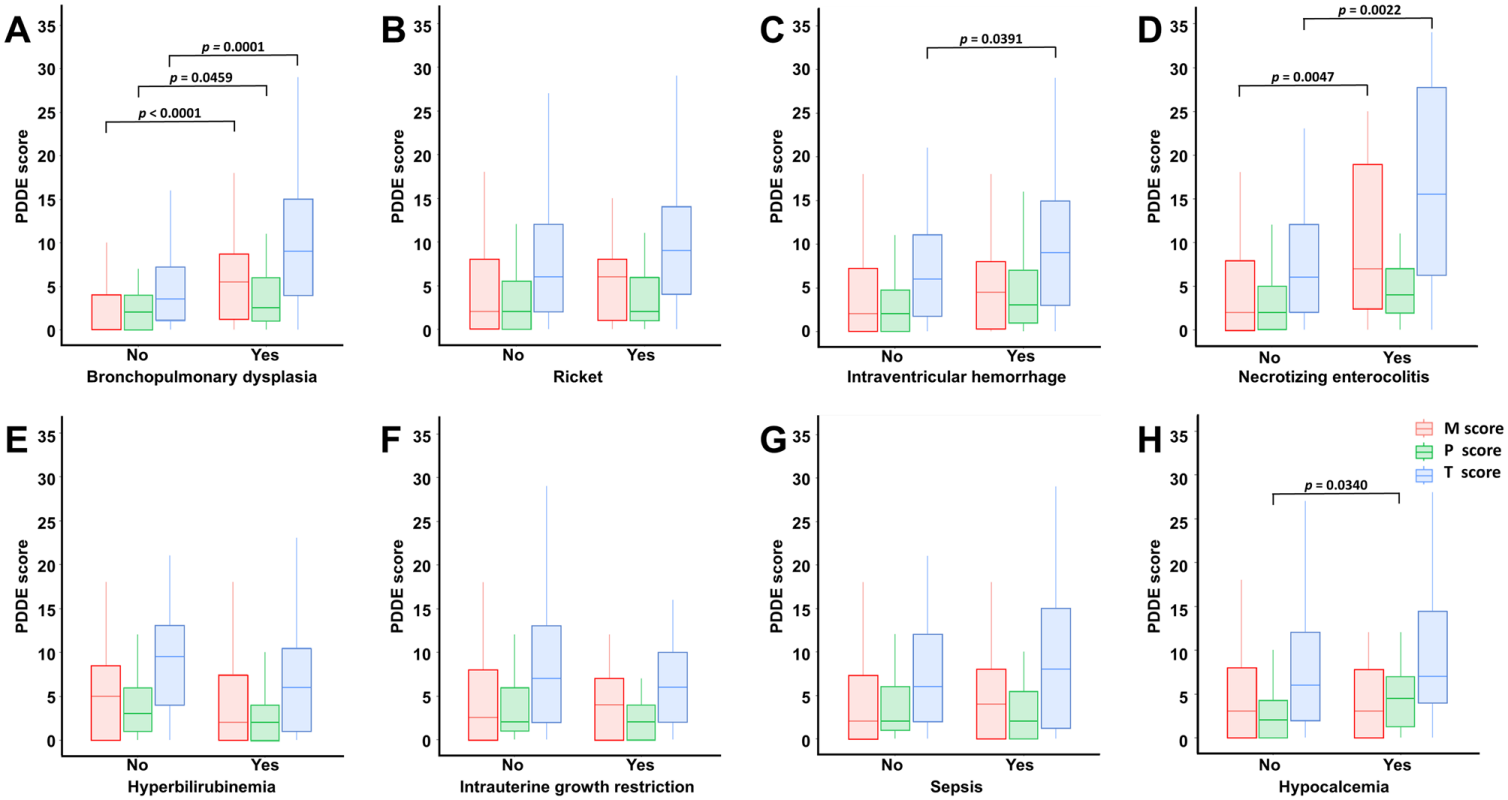
Preterm developmental defects of enamel (PDDE) scores based on medical complications. Preterm infants with bronchopulmonary dysplasia had significantly higher M (p < 0.0001), P (p = 0.0459), and T (p < 0.0001) scores than those who were not diagnosed. The T-score was significantly higher in preterm infants with intraventricular hemorrhage than that in those who were not diagnosed (p = 0.0391). Preterm infants with necrotizing enterocolitis had significantly higher M (p = 0.0047) and T (p = 0.0022) scores than those without necrotizing enterocolitis. Finally, preterm infants with hypocalcemia had significantly higher P (p = 0.0340) score than those without hypocalcemia. *M score* assessment of hypomineralization in qualitative defects; *P score* assessment of hypoplasia in quantitative defects.

Preterm infants with BPD had significantly higher M (hypomineralization, p < 0.0001), P (hypoplasia, p = 0.0459), and T (M + P, p = 0.0001) scores than those without BPD. The T score of preterm infants with IVH was significantly higher than in those without IVH (p = 0.0391). Preterm infants with NEC had significantly higher M (p = 0.0047) and T (p = 0.0022) scores than those without NEC. Preterm infants with hypocalcemia had significantly higher P (p = 0.0340) than those without hypocalcemia (Fig. 1).

### Regression analysis for severity of DDE in preterm infants

No prenatal factors were associated with increasing severity of DDEs. Preterm infants with GA < 28 weeks had higher M (*β* = 5.1274, p = 0.0050), P (*β* = 1.4425, p = 0.0342), and T (*β* = 6.4797, *p* = 0.0017) scores than those born after 28 weeks of gestation. Infants with BW < 1000 g had elevated M (*β* = 3.1861, p = 0.0788), P (*β* = 1.4168, p = 0.0343), and T (*β* = 4.3917, *p* = 0.0320) scores. The 1- and 5-min APGAR scores minimally affected M; however, they were risk factors for the P score. BPD and NEC are medical complications that raised M (*β* = 6.2045, p = 0.0003), P (*β* = 1.5417, p = 0.0237), and T (*β* = 7.6496, p < 0.0001) scores for BPD, and significantly increased M (*β* = 12.6519, p < 0.0001), P (*β* = 2.4692, p = 0.0196), and T (*β* = 14.9726, p < 0.0001) scores for NEC. IVH elevated P (*β* = 1.4622, p = 0.0384) and T (*β* = 4.9561, p = 0.0166) scores. NICU admissions > 2 months and intubation > 1 month increased PDDE scores (Table 3 and Supplementary Tables S1 and S2). Based on Tables 2 and 3, with regard to natal factors, the power is 0.89 or higher based on a sample size of 118 individuals and a significance level (alpha) of 0.05.

**Table 3:** Regression analysis of potential risk factors for increasing the preterm developmental defects of enamel score.

T score (M + P score)		Descriptive analysis						Regression analysis		
Independent variable		n	Average	SD	Median	IQR	P-value	B	95% CI	P-value
Prenatal risk factors										
Advanced maternal age	Yes	59	10.46	10.86	7.00	13.00	0.0795	3.5041	− 0.3978 to 7.4060	0.0778
	No	43	6.95	8.14	4.00	10.00		Reference		
Maternal abortion history	Yes	27	11.07	12.51	7.00	14.00	0.6173	3.1241	− 1.2933 to 7.5415	0.1633
	No	60	7.95	7.97	6.50	9.50		Reference		
Neonatal risk factors										
Gestational age (weeks)	< 28	43	14.09	13.31	10.00	13.00	0.0047*	6.4797	2.4966–10.4628	0.0017*
	≥ 28	75	7.61	8.52	5.00	12.00		Reference		
Birth weight (g)	< 1000	47	12.62	11.60	10.00	12.00	0.0100*	4.3917	0.3851–8.3982	0.0320*
	≥ 1000	71	8.23	10.17	5.00	12.00		Reference		
Delivery mode	c-sec	97	10.42	11.05	7.00	13.00	0.2051	2.5227	− 7.8629 to 2.8175	0.3514
	NVSD	20	7.90	10.63	3.00	9.50		Reference		
APGAR score 1 min^a^	< 7	95	10.11	10.55	7.00	13.00	0.0029*	6.9386	0.7702–13.1070	0.0278*
	≥ 7	12	3.17	5.70	0.00	3.50		Reference		
APGAR score 5 min^a^	< 8	84	10.26	9.72	8.00	11.50	0.0005*	4.3489	− 0.4268 to 9.1245	0.0738
	≥ 8	23	5.91	11.99	2.00	4.00		Reference		
Multiple pregnancy	Multiplet	44	9.66	12.42	6.00	12.00	0.2821	-0.5327	− 4.6975 to 3.6321	0.8005
	singlet	73	10.19	10.09	8.00	13.00		Reference		
Postnatal risk factors										
Medical complications										
Bronchopulmonary dysplasia	Yes	66	12.88	12.14	10.00	11.00	0.0001*	7.6496	3.9083 to 11.3910	< 0.0001*
	No	48	5.23	5.68	3.50	6.50		Reference		
Ricket	Yes	27	12.44	13.00	9.00	11.00	0.1591	3.6513	− 0.9505 to 8.2532	0.1187
	No	87	8.79	9.68	7.00	11.00		Reference		
Intraventricular hemorrhage	Yes	40	12.88	12.60	9.00	13.00	0.0391*	4.9561	0.9168–8.9954	0.0166*
	No	74	7.92	8.98	6.00	10.00		Reference		
Necrotizing enterocolitis	Yes	13	22.92	16.18	27.00	27.00	0.0022*	14.9726	9.4170–20.5282	< 0.0001*
	No	101	7.95	8.37	7.00	10.00		Reference		
Hyperbilirubinemia	Yes	73	8.44	10.04	6.00	11.00	0.1018	-3.3909	− 7.4633 to 0.6815	0.1018
	No	41	11.83	11.36	10.00	11.00		Reference		
Intrauterine growth restriction	Yes	21	6.24	5.45	6.00	8.00	0.2111	-4.3397	− 9.4535 to 0.7742	0.0954
	No	90	10.58	11.50	7.50	13.00		Reference		
Hypocalcemia	Yes	18	10.28	9.35	7.00	11.00	0.5090	0.7361	− 4.6864 to 6.1587	0.7884
	No	96	9.54	10.87	7.00	11.50		Reference		
Sepsis	Yes	31	10.74	10.95	9.00	14.00	0.5882	1.4889	− 2.9476 to 5.9254	0.5074
	No	83	9.25	10.52	7.00	11.00		Reference		
Parenteral feeding (month)^a^	≥ 1	28	12.14	11.28	9.00	10.00	0.0351*	4.3400	− 0.0891 to 8.7692	0.0547
	< 1	71	7.80	9.46	5.00	12.00		Reference		
Endotracheal intubation (month)^a^	≥ 1	23	13.43	13.12	9.00	10.00	0.0426*	5.0583	0.2779–9.8387	0.0383*
	< 1	85	8.38	9.36	6.00	12.00		Reference		
NICU admission (month)^a^	≥ 2	69	12.20	10.58	10.00	11.00	< 0.0001*	7.3458	3.5820–11.1095	0.0002*
	< 2	42	4.86	7.35	2.00	7.00		Reference		

For descriptive analysis, the *p*-value was tested using the Wilcoxon rank-sum test. For logistic regression, the *p*-value was tested using a generalized linear model.

*C-sec* cesarean section, *NSVD* normal spontaneous vaginal delivery, *NICU* neonatal intensive care unit.

*p < 0.05 indicates statistical significance.

^a^Indicates that the cutoff value was determined using the receiver operating characteristic (ROC) curve.

### DDE distribution

**Figure 2:**
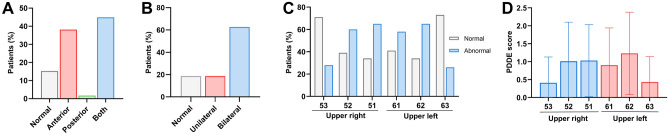
Distribution of developmental defects of enamel (DDE). Forty-four infants (43.1%) had DDE affecting only the primary incisors, and 55 (53.9%) had defects in both primary incisors and molars, indicating a significantly higher proportion than that of preterm infants with defect affecting only the primary molars (1.69%). DDE primarily occurred bilaterally in the anterior maxillary region (62.71% of cases). The prevalence of DDE and PDDE scores in both maxillary primary central and lateral incisors was significantly higher than that in primary canine.

Of the infants, 44 (43.1%) had DDE affecting only the primary incisors, and 55 (53.9%) had both primary incisors and molars, indicating a significantly higher proportion than preterm infants affecting only the primary molars (1.69%). DDE primarily occurred bilaterally in the maxillary anterior region (62.71% of cases). The prevalence of DDE and PDDE scores in both maxillary primary central and lateral incisors was significantly higher than that in primary canines (Fig. 2).

### First primary tooth eruption in preterm infants

The primary tooth eruption in preterm groups was confirmed as follows: extremely preterm 8.07 ± 3.46, very preterm 8.58 ± 3.22, and late preterm 7.28 (± 2.73) months. No significant correlation was found between the gestational and eruption age (Table 1). However, as GA decreased, corrected age (CA) increased. Extremely preterm, very preterm, and moderate to late preterm groups showed the following CA: 12.17 ± 5.48, 11.16 ± 3.35, and 8.40 ± 2.61; Table 1).

## Discussion

This study examined factors affecting amelogenesis disruption leading to DDE in preterm infants. The primary objective of this paper is to identify factors impacting the prevalence and severity of DDE, demanding early dental examination and intervention in high-risk preterm infants. Distinguishing itself from previous research, this study not only explores the prevalence of DDE based on natal factors but also introduces a PDDE index to assess the severity of DDE. The PDDE index was developed by modifying the previously used DDE Index proposed in 1989 and applying it to preterm deciduous teeth. According to the PDDE index proposed in this study, quantitative defects of enamel were scored as P scores, while qualitative defects were scored as M scores. The quantitative and qualitative defects of enamel are induced by different processes during amelogenesis. By evaluating them separately with their respective scores, it becomes possible to investigate which amelogenesis stages are more influenced by natal factors.

Results were similar to that of previous studies, indicating a significant increase in the prevalence and severity of DDE in preterm infants, with a GA < 28 weeks and a BW < 1000 g. This suggested the importance of calcium accumulation after 28 weeks of gestation for amelogenesis, significantly affecting both the maturation and secretion phases^25^. Fetal serum calcium increases exponentially between the GA of 24 and 37 weeks, contributing to 80% of the required mineral accumulation in the third trimester^26^. Consequently, the high prevalence and severity of DDE in preterm infants born before calcium accumulation is expected. This aligns with prior studies stating that DDE prevalence ranges from 46 to 96%, with shorter GA and lower BW associated with a higher prevalence^14,15,27,28^. Notably, this study confirms an increase in DDE severity compared to previous research.

Advanced maternal age increased the prevalence of DDE, however, it does not appear to affect severity. This implied that although advanced maternal age is associated with an increased risk of preterm delivery^29^, it is difficult to conclude that senescence directly disturbs amelogenesis by raising the severity of DDE. Only a correlation between young maternal age and the prevalence of DDE was confirmed; however, not with advanced maternal age^4,27,30^. Given the growing trend of advanced maternal age, further research into the relationship between the health status of older mothers and DDE is warranted.

APGAR scores, a straightforward assessment method for newborns, evaluate skin color, heart rate, muscle tone, respiration, and reflexes immediately after birth^31^. In this study, 1-min APGAR scores ≥ 7 and 5-min scores ≥ 8 were associated with reduced DDE prevalence and severity, consistent with normal APGAR scores ranging from 7 to 10^31^. This indicated that the APGAR score serves as an indicator of infant health and a predictive criterion for DDE occurrence and severity.

Common diseases in premature infants include BPD, rickets, IVH, NEC, hyperbilirubinemia, IUGR, hypocalcemia, and sepsis^1,3,32^. Although these complications did not affect the prevalence of DDE, BPD, IVH, NEC, and hypocalcemia increased its severity. In other words, there was no significant difference in the prevalence of DDE between preterm infants with and without complications; however, there was a significant difference in severity. This suggested that the presence of complications does not necessarily lead to the development of DDE; however, it may exacerbate pre-existing DDE. Complications that disrupted enamel maturation included BPD and NEC, whereas those that affected enamel secretion included BPD, IVH, NEC, and hypocalcemia.

BPD, characterized by inadequate spontaneous breathing, often requires oxygen supplementation with endotracheal intubation. Intubation before tooth eruption can cause localized trauma to developing teeth^33^. Additionally, BPD directly affects mineral metabolism and reduces bone mineral content owing to drug treatment, potentially leading to DDE^19,34^. IVH is diagnosed when bleeding occurs in the germinal matrix beneath the ependyma of the brain ventricles, and can lead to neurological impairments^22,35^. Neurological abnormalities increase the incidence of DDE, and systemic disorders affecting neurodevelopment can impact amelogenesis^22–24^. NEC, a disease causing intestinal or colon necrosis in newborns often requiring surgical resection of the affected bowel, reduces the total absorptive surface area of the intestine, limiting mineral absorption^20,21^. Hypocalcemia, which results in a low concentration of crucial minerals during amelogenesis, leads to DDE. Postnatal factors such as the duration of endotracheal intubation and NICU admission significantly increase the PDDE score, aligning with previous research^27^. It is common for preterm infants to be admitted to the NICU for airway management competency with endotracheal intubation immediately after birth.

Localized trauma can result in defects in the affected area, which aligns with the findings of our study of a higher prevalence and severity of DDE in the anterior teeth, consistent with previous research^30,36–38^. Notably, localized trauma from tracheal intubation induces this effect on primary incisors. In addition, premature birth can lead to incomplete calcification of the primary incisors as enamel formation of the teeth begins at approximately 4–5 months in the uterus, at the earliest. However, some studies have shown a high frequency of DDE in the maxillary first primary molars^38,39^, emphasizing the need for further studies on the prevalence of DDE in each tooth.

The timing of tooth eruption is a key indicator of overall growth and development^40^. This study confirmed that as GA and BW decreased, the CA of the first primary tooth eruption was delayed, particularly in extremely preterm infants and those with BW < 1000 g infants. This implied that dental development rates in preterm infants are influenced by GA and BW, similar to their impact on other aspects of physical and cognitive development. Additionally, using CA instead of chronological age for dental development rate comparison is reasonable, especially in children aged three or younger^41,42^. These findings differ from those of prior research, which showed a correlation between tooth eruption age and GA when calculated chronologically; however, not when adjusted using CA^43^. The average GA of the preterm infants examined in previous studies was 36.5 weeks, and they were classified as moderate to late preterm infants. In contrast, this study investigated CA in extremely to late preterm infants, with a significant delay in CA among extremely preterm infants born before the exponential calcium accumulation^26^.

This study had several limitations. First, there were no comparisons between preterm and full-term infants, owing to difficulties in assembling a control group of full-term infants with common preterm birth complications. Second, the PDDE relies heavily on the visual inspection of the examiner, making it difficult to maintain objectivity. To mitigate this, we attempted to enhance the consistency of dental color assessment by capturing intraoral photographs of the infants during each visit and comparing them over time. Finally, gestational age as main variable was correlated with all variables, including birth weight, APGAR score, BPD, and NEC. Therefore, a univariate analysis was performed and a risk model through multivariate analysis could not be presented.

The strengths of the study included a well-documented cohort of preterm infants with medical and dental records, enabling simultaneous investigation of the relationship between various natal factors and DDE. Regular check-ups excluded post-eruption influences such as dental caries, enhancing DDE assessment reliability. Additionally, it proposed quantitative criteria for evaluating DDE, enabling the assessment of factor impact during the secretory and maturation phases. This study examined the factors affecting DDE severity and distribution in preterm infants, emphasizing esthetic concerns, tooth sensitivity, and caries risk, highlighting the importance of early dental intervention.

## Material and methods

### Patient selection

The cohort was composed of 178 preterm infants who visited the Department of Pediatric Dentistry of Yonsei University Dental Hospital between 2019 and 2022. Most of them had been admitted to the NICU at Severance Hospital, Yonsei University College of Medicine. After discharge, dental examinations were performed through the consultation system from the Neonatology Department of the same hospital. The inclusion and exclusion criteria were as follows:

Inclusion criteria:

1. Premature infants who visited the Department of Pediatric Dentistry before the eruption of primary teeth was completed.
2. Premature infants who had been admitted to NICU.
3. Patients who underwent regular dental check-ups at intervals of 1–6 months.

Exclusion criteria:

- Patients with a history of trauma that could hinder accurate PDDE scoring.
- Patients who developed dental caries before the evaluation of their enamel defects, making it difficult to calculate an accurate PDDE score.
- Patients with < 20 evaluable primary teeth due to early extraction or congenital missing teeth.

Sixty infants were excluded based on the exclusion criteria, and 118 preterm infants were included in the study. Written informed consent for participation in the study was obtained from parents of preterm infants. This study was approved by the Institutional Review Board of Yonsei University Dental Hospital (IRB no. 2-2019-0045).

### Preterm developmental defects of enamel index

This study quantitatively assessed the DDE of preterm The timing of tooth eruption is a key indicator of overall growth and development^40^. This study confirmed that as GA and BW decreased, the CA of the first primary tooth eruption was delayed teeth based on the PDDE index (Table 4), which was devised by revising the DDE index proposed by Clarkson^10^ and the Modified DDE Index^44^. The PDDE index categorizes DDE as enamel hypoplasia or hypomineralization, assigning P and M scores to severity. Enamel hypoplasia was assessed based on the extent of hard tissue defects as follows: normal tooth morphology (0 points), structural defects confined to the enamel (1 point), and defects affecting both enamel and dentin (2 points). The sum of the scores for the 20 primary teeth was defined as the P score. Enamel hypomineralization was graded according to tooth color and opacity as follows: normal (0 points), white opacity (1 point), yellowish (2 points), and yellowish to brownish (3 points). The sum of the scores of all 20 primary teeth was defined as the M score. The final T score was the sum of the P and M scores.

**Table 4:** Classification of developmental defects of enamel using the preterm developmental defects of enamel (PDDE) index.

Characteristics	Example	PDDE score
Qualitative defects: Opacities with abnormal color shades		M score
Normal color and opacity		0
White opacity defect		1
Yellow defect		2
Yellow–brown defect		3
Quantitative defects: Structural defect associated with enamel hypoplasia		P score
Normal tooth morphology		0
Dental structural defects confined to enamel		1
Dental structural defects that affect both enamel and dentin		2

### Variables

The independent variables were classified into three categories: prenatal factors (maternal age, paternal age, and maternal abortion history), neonatal factors (sex, GA, BW, APGAR score, delivery mode, and single or multiple pregnancy status), postnatal variables (duration of NICU hospitalization, parenteral feeding, and endotracheal intubation), and medical complications (BPD, rickets, IVH, NEC, hyperbilirubinemia, sepsis, IUGR, and hypocalcemia).

Advanced maternal age was defined as ≥ 35 years at delivery^45^. APGAR scores were measured 1 min and 5 min after birth, referred to as 1-min and 5-min APGAR scores, respectively. The groups were categorized based on a 7-point and 8-point cutoff for the 1-min and 5-min APGAR scores, respectively, with each group demonstrating statistically significant differences. These variables were investigated by reviewing the NICU admission, discharge, and birth records of preterm infants. The chronological age and CA were investigated in this study. Chronological age was calculated based on the actual date of birth, whereas CA was calculated based on the expected date of birth. CA is defined as follows: CA = chronological age − (period of time born before the expected birth date).

The DDE was evaluated based on the PDDE index. Intraoral photographs of newly erupted teeth and dental records were documented when the preterm infants visited the Department of Pediatric Dentistry for oral examination. Initial PDDE scores were assigned to existing teeth and new teeth were scored during follow-up checkups. When dental caries was suspected, radiographs or quantitative light-induced fluorescence was used to distinguish DDE from dental caries.

### Statistical analyses

Descriptive statistics were used for data analysis. The Chi-squared and Kruskal–Wallis tests were used to assess the association between GA and natal factors. Bonferroni post-hoc tests were used to conduct multiple pairwise comparisons after the initial analyses. Regression analysis using Firth’s method was employed to evaluate the factors that may influence the prevalence of DDE. Regression analysis with a generalized linear model was performed to assess factors that may increase the PDDE score. The cutoff point was determined using a receiver operating characteristic curve. Descriptive statistics and statistical analyses were conducted using SAS (version 9.4; SAS Institute, Inc., Cary, NC, USA) and R (version 4.0.0; R Foundation for Statistical Computing, Vienna, Austria) software. The minimum significance level adopted was 5% (0.05).

### Ethics declarations

The study was conducted in accordance with the Declaration of Helsinki, and was approved by the Institutional Review Board of Yonsei University Dental Hospital in Seoul, Korea (IRB no. 2-2019-0045).

### Supplementary Information

Supplementary Tables.

## Data Availability

The data used in this manuscript are available to the editors upon request from the corresponding authors.
