# Supplementary material for: Natal factors affecting developmental defects of enamel in preterm infants: a prospective cohort study
Source: Sci Rep. 2024 Jan 24;14:2089. doi: 10.1038/s41598-024-52525-2 (PMC10808204; doi:10.1038/s41598-024-52525-2)
Supplement: Supplementary file 1 — Supplementary Tables. [file 41598_2024_52525_MOESM1_ESM.docx]

**Supplementary Table S1.** Regression analysis of potential risk factors for increasing M score

| **M score** | | |  | **Descriptive analysis** | | | | | | **Regression analysis** | | |
| --- | --- | --- | --- | --- | --- | --- | --- | --- | --- | --- | --- | --- |
| **Independent variable** | | |  | **n** | **Average** | **SD** | **Median** | **IQR** | **P-value** | **B** | **95% CI** | **P-value** |
| **Prenatal risk factors** | | | | | | | | | | | | |
| Advanced maternal age | | | Yes | 59 | 6.83 | 9.34 | 4.00 | 9.00 | 0.0499* | 3.0631 | -0.1673 - 6.2934 | 0.0628 |
|  | | | No | 43 | 3.77 | 6.05 | 2.00 | 6.00 |  | Reference | | |
| Maternal abortion history | | | Yes | 27 | 5.05 | 8.24 | 2.00 | 8.00 | 0.3729 | 2.9963 | -0.8007 - 6.7933 | 0.1204 |
|  | | | No | 60 | 4.63 | 6.71 | 3.00 | 7.00 |  | Reference | | |
| **Neonatal risk factors** | | | | | | | | | | | | |
| Gestational age (weeks) | | < 28 | | 43 | 9.77 | 12.23 | 6.00 | 8.00 | 0.0026* | 5.1274 | 1.5820 - 8.6729 | 0.0050* |
|  | | ≥28 | | 75 | 4.64 | 7.24 | 2.00 | 7.00 |  | Reference | | |
| Birth weight (g) | < 1000 | | | 47 | 8.43 | 10.47 | 6.00 | 9.00 | 0.0116* | 3.1861 | -0.3726 - 6.7448 | 0.0788 |
|  | ≥1000 | | | 71 | 5.24 | 8.90 | 2.00 | 7.00 |  | Reference | | |
| Delivery mode | c-sec | | | 97 | 6.86 | 9.95 | 4.00 | 8.00 | 0.2999 | 1.8057 | -2.9091 - 6.5204 | 0.4496 |
|  | NVSD | | | 20 | 5.05 | 8.24 | 2.00 | 8.00 |  | Reference | | |
| APGAR score 1min ^a^ | | | < 7 | 95 | 6.35 | 9.02 | 4.00 | 9.00 | 0.0191* | 3.9307 | -1.3652 - 9.2266 | 0.1441 |
|  | | | ≥7 | 12 | 2.42 | 5.45 | 0.00 | 1.50 |  | Reference | | |
| APGAR score 5min ^a^ | | | < 8 | 84 | 6.23 | 8.11 | 4.00 | 9.00 | 0.0067* | 1.4871 | -2.6127 - 5.5868 | 0.4736 |
|  | | | ≥8 | 23 | 4.74 | 10.97 | 0.00 | 2.00 |  | Reference | | |
| Multiple pregnancy | Multiplet | | | 44 | 6.45 | 10.92 | 2.50 | 8.00 | 0.3485 | -0.1482 | -3.8214 - 3.5250 | 0.9364 |
|  | singlet | | | 73 | 6.60 | 8.92 | 4.00 | 9.00 |  | Reference | | |
| **Postnatal risk factors** | | | | | | | | | | | | |
| Medical complications | | | | | | | | | | | | |
| Bronchopulmonary dysplasia | | | Yes | 66 | 8.79 | 11.02 | 6.00 | 8.00 | <0.0001* | 6.2045 | 2.9181 - 9.4910 | 0.0003* |
|  | | | No | 48 | 2.58 | 3.77 | 0.00 | 4.00 |  | Reference | | |
| Ricket | | | Yes | 27 | 8.59 | 11.28 | 6.00 | 9.00 | 0.0691 | 3.1673 | -0.8370 - 7.1716 | 0.1199 |
|  | | | No | 87 | 5.43 | 8.43 | 2.00 | 8.00 |  | Reference | | |
| Intraventricular hemorrhage | | | Yes | 40 | 8.20 | 10.88 | 5.50 | 9.00 | 0.0927 | 3.1189 | -0.4395 - 6.6774 | 0.0852 |
|  | | | No | 74 | 5.08 | 8.08 | 2.00 | 8.00 |  | Reference | | |
| Necrotizing enterocolitis | | | Yes | 13 | 17.38 | 16.23 | 10.0 | 21.0 | 0.0047* | 12.6519 | 7.7832 - 17.5207 | <0.0001* |
|  | | | No | 101 | 4.73 | 6.80 | 2.00 | 8.00 |  | Reference | | |
| Hyperbilirubinemia | | | Yes | 73 | 5.37 | 8.48 | 2.00 | 8.00 | 0.1120 | -2.2399 | -5.8014 - 1.3216 | 0.2153 |
|  | | | No | 41 | 7.61 | 10.39 | 5.00 | 9.00 |  | Reference | | |
| Intrauterine growth restriction | | | Yes | 21 | 3.76 | 3.94 | 4.00 | 7.00 | 0.3733 | -3.0714 | -7.5419 - 1.3990 | 0.1761 |
|  | | | No | 90 | 6.83 | 10.13 | 3.00 | 9.00 |  | Reference | | |
| Hypocalcemia | | | Yes | 18 | 5.11 | 6.44 | 3.00 | 8.00 | 0.9306 | -1.2639 | -5.9776 - -3.4498 | 0.5963 |
|  | | | No | 96 | 6.83 | 10.39 | 5.00 | 9.00 |  | Reference | | |
| Sepsis | | | Yes | 31 | 7.16 | 9.17 | 4.00 | 10.00 | 0.3392 | 1.3541 | -2.5054 - 5.2135 | 0.4884 |
|  | | | No | 83 | 5.81 | 9.29 | 3.00 | 8.00 |  | Reference | | |
| Parenteral feeding (month) ^a^ | | | ≥ 1 | 28 | 7.79 | 9.52 | 4.00 | 8.50 | 0.0546 | 2.8280 | -0.9390 - 6.4939 | 0.1290 |
|  | | | < 1 | 71 | 4.96 | 7.74 | 2.00 | 8.00 |  | Reference | | |
| Endotracheal intubation (month) ^z^ | | | ≥ 1 | 23 | 8.52 | 12.59 | 4.00 | 9.00 | 0.2558 | 3.2276 | -0.8662 - 7.3215 | 0.1210 |
|  | | | < 1 | 85 | 5.29 | 7.48 | 2.00 | 8.00 |  | Reference | | |
| NICU admission (month) ^z^ | | | ≥ 2 | 69 | 7.90 | 9.70 | 6.00 | 9.00 | <0.0001* | 5.2081 | 1.9404 - 8.4757 | 0.0020* |
|  | | | < 2 | 42 | 2.69 | 5.71 | 0.00 | 4.00 |  | Reference | | |

For descriptive analysis, the *p*-value was tested using the Wilcoxon rank-sum test. For logistic regression, the *p*-value was tested using a generalized linear model. ^*^ *p* <0.05 indicates statistical significance, ^a^ indicates that the cutoff value was determined using the receiver operating characteristic (ROC) curve.

*C-sec* cesarean section; *NSVD* normal spontaneous vaginal delivery; *NICU* neonatal intensive care unit.

**Supplementary Table S2.** Regression analysis of potential risk factors for increasing P score

| **P score** | | |  | **Descriptive analysis** | | | | | | **Regression analysis** | | |
| --- | --- | --- | --- | --- | --- | --- | --- | --- | --- | --- | --- | --- |
| **Independent variable** | | |  | **n** | **Average** | **SD** | **Median** | **IQR** | **P-value** | **B** | **95% CI** | **P-value** |
| **Prenatal risk factors** | | | | | | | | | | | | |
| Advanced maternal age | | | Yes | 59 | 3.47 | 3.82 | 2.00 | 5.00 | 0.4308 | 0.3585 | -1.1276 - 1.8442 | 0.6334 |
|  | | | No | 43 | 3.12 | 3.61 | 2.00 | 4.00 |  | Reference | | |
| Maternal abortion history | | | Yes | 27 | 3.11 | 3.95 | 2.00 | 5.00 | 0.4957 | -0.1556 | -1.7124 - 1.4013 | 0.8430 |
|  | | | No | 60 | 3.27 | 3.09 | 2.00 | 5.00 |  | Reference | | |
| **Neonatal risk factors** | | | | | | | | | | | | |
| Gestational age (weeks) | | < 28 | | 43 | 4.26 | 4.44 | 2.00 | 6.00 | 0.0960 | 1.4425 | 0.1097 – 2.7753 | 0.0342* |
|  | | ≥28 | | 75 | 2.81 | 2.86 | 2.00 | 4.00 |  | Reference | | |
| Birth weight (g) | < 1000 | | | 47 | 4.19 | 4.40 | 2.00 | 6.00 | 0.1201 | 1.4168 | 0.1066 - 2.7271 | 0.0343* |
|  | ≥1000 | | | 71 | 2.77 | 2.79 | 2.00 | 4.00 |  | Reference | | |
| Delivery mode | c-sec | | | 97 | 3.41 | 3.45 | 2.00 | 5.00 | 0.1714 | -0.5624 | -2.3075 - 1.1827 | 0.5245 |
|  | NVSD | | | 20 | 2.85 | 4.23 | 1.50 | 3.00 |  | Reference | | |
| APGAR score 1min | | | < 7 | 95 | 3.63 | 3.70 | 2.00 | 5.00 | 0.0021* | 2.8816 | 0.7438 - 5.0193 | 0.0087* |
|  | | | ≥7 | 12 | 0.75 | 1.14 | 0.00 | 2.00 |  | Reference | | |
| APGAR score 5min | | | < 8 | 84 | 3.89 | 3.78 | 3.00 | 5.00 | 0.0003* | 2.7189 | 1.1055 - 4.3324 | 0.0012* |
|  | | | ≥8 | 23 | 1.17 | 1.80 | 0.00 | 2.00 |  | Reference | | |
| Multiple pregnancy | Multiplet | | | 44 | 3.07 | 3.23 | 2.00 | 4.00 | 0.7030 | -0.3976 | -1.7542 - 0.9591 | 0.5627 |
|  | singlet | | | 73 | 3.47 | 3.79 | 2.00 | 5.00 |  | Reference | | |
| **Postnatal risk factors** | | | | | | | | | | | | |
| Medical complications | | | | | | | | | | | | |
| Bronchopulmonary dysplasia | | | Yes | 66 | 4.00 | 4.05 | 2.50 | 5.00 | 0.0459* | 1.5417 | 0.2100 -2.8733 | 0.0237* |
|  | | | No | 48 | 2.46 | 2.68 | 2.00 | 4.00 |  | Reference | | |
| Ricket | | | Yes | 27 | 3.85 | 4.37 | 2.00 | 5.00 | 0.5716 | 0.6564 | -0.9212 - 2.2341 | 0.4114 |
|  | | | No | 87 | 3.20 | 3.35 | 2.00 | 6.00 |  | Reference | | |
| Intraventricular hemorrhage | | | Yes | 40 | 4.30 | 4.42 | 3.00 | 6.00 | 0.0880 | 1.4622 | 0.0793 - 2.8450 | 0.0384* |
|  | | | No | 74 | 2.84 | 3.00 | 2.00 | 5.00 |  | Reference | | |
| Necrotizing enterocolitis | | | Yes | 13 | 5.54 | 5.44 | 4.00 | 5.00 | 0.0720 | 2.4692 | 0.4037 - 4.5346 | 0.0196* |
|  | | | No | 101 | 3.07 | 3.23 | 2.00 | 5.00 |  | Reference | | |
| Hyperbilirubinemia | | | Yes | 73 | 2.90 | 3.21 | 2.00 | 4.00 | 0.0966 | -1.2422 | -2.6247 - 0.1402 | 0.0777 |
|  | | | No | 41 | 4.15 | 4.15 | 3.00 | 5.00 |  | Reference | | |
| Intrauterine growth restriction | | | Yes | 21 | 2.48 | 2.36 | 2.00 | 4.00 | 0.3843 | -1.1349 | -2.8787 - 0.6088 | 0.1998 |
|  | | | No | 90 | 3.61 | 3.86 | 2.00 | 5.00 |  | Reference | | |
| Hypocalcemia ^a^ | | | Yes | 18 | 5.00 | 4.54 | 4.50 | 6.00 | 0.0340* | 1.9583 | 0.1502 - 3.7665 | 0.0340* |
|  | | | No | 96 | 3.04 | 3.35 | 2.00 | 4.50 |  | Reference | | |
| Sepsis | | | Yes | 31 | 3.29 | 3.84 | 2.00 | 6.00 | 0.6477 | -0.0832 | -1.5950 - 1.4287 | 0.9134 |
|  | | | No | 83 | 3.37 | 3.54 | 2.00 | 5.00 |  | Reference | | |
| Parenteral feeding (month) ^b^ | | | ≥ 1 | 28 | 4.36 | 3.91 | 3.50 | 5.50 | 0.0197* | 1.6811 | 0.1259 - 3.2363 | 0.0344* |
|  | | | < 1 | 71 | 2.68 | 3.35 | 2.00 | 4.00 |  | Reference | | |
| Endotracheal intubation (month) ^b^ | | | ≥ 1 | 23 | 4.78 | 3.97 | 4.00 | 5.00 | 0.0125* | 1.8414 | 0.1573 - 3.5256 | 0.0324* |
|  | | | < 1 | 85 | 2.34 | 3.52 | 2.00 | 5.00 |  | Reference | | |
| NICU admission (month) ^b^ | | | ≥ 2 | 69 | 4.13 | 4.06 | 3.00 | 5.00 | 0.0091* | 1.9638 | 0.5918 - 3.3358 | 0.0054* |
|  | | | < 2 | 42 | 2.17 | 2.44 | 2.00 | 3.00 |  | Reference | | |

For descriptive analysis, the *p*-value was tested using the Wilcoxon rank-sum test. For logistic regression, the *p*-value was tested using a generalized linear model. ^*^ *p* <0.05 indicates statistical significance, ^a^ indicates that the cutoff value was determined using the receiver operating characteristic (ROC) curve.

*C-sec* cesarean section; *NSVD* normal spontaneous vaginal delivery; *NICU* neonatal intensive care unit.
